# Organization of the Ohio State Dental Association

**Published:** 1866-08

**Authors:** 


### Editorial.

### ORGANIZATION OF THE OHIO STATE DENTAL ASSOCIATION.

Persuant to a call signed by a number of the profession, and issued about the 1st of June, quite a large number of the profession assembled in Columbus on Tuesday the 26th of June, for the purpose of organizing a State Dental Society. The assemblage was for the most part composed of the earnest workers of the profession. They seemed to be thoroughly impressed that a great responsibility rested upon them. They were about to attempt a work which if harmoniously and well done, would result in great good, would exercise a wide and lasting influence.

In the temporary organization, Dr. B. Strickland of Cleveland, was chosen chairman, Dr. H. A. Smith of Cincinnati, Secretary, and Dr. B. T. Spelman, Treasurer. A committee was appointed to prepare and present a constitution and by-laws. That committee reported a draft that with a very few slight modifications, was entirely satisfactory to the meeting. This constitution and by-laws were adopted, and then upon motion it agreed that only those who were active members of some local society in the state, or graduates of a dental college, were elegible to membership in the organization. All thus qualified, proceeded to sign the constitution and pay the initiation fee. The officers for the permanent organization were then elected and installed; a committee on membership was appointed who then proceeded to receive and examine applicants for membership, who were not eligible in the organization, quite a large list of names were presented by the committee, all of whom so far as we know were elected members. We think, there was a strong disposition apparent, though not ostentatiously so, to make merit and ability the basis of membership. This we hope may serve as a wholesome stimulus upon some.

There were several topics of general interest taken up and discussed ; among which was the proposed bill to regulate the practice of Dentistry in the State of Ohio. Upon this subject, after a free examination and discussion, there was but one opinion, that it was entirely feasible and greatly to be desired, both upon the part of the people and the profession; and there was a unanimous resolution to labor earnestly for its procurement by all proper effort. The fact was pretty clearly brought out that the character of the opposition hitherto made against it was of a very questionable character, especially in the method of its procedure.

Other subjects were discussed and elicited much interest. The interest and earnestness that was manifested will be appreciated when we consider that there was three long sessions held each day, and at the final adjournment it was agreed to hold the next meeting, which will be in January next, during three days.

One work was performed that has not been attempted by any other dental society in existence so far as we know, and that was the drafting and adopting a code of Dental Ethics. It has often occurred to us as a matter of wonder, that dental societies have not framed and adopted such an instrument; for by it certainly a great many things that have been harrasing and vexing, in associations, would have been set at rest; we think all societies would find such an instrument highly beneficial. It will be published with the proceedings of the society, and we ask for it a careful perusal and consideration. The society has upon its roll the names of forty members; thus entitling it to eight delegates, in the American Dental Association, which were appointed. There will be two meetings annually; the annual meeting in January, and the semi-annual in June.

The officers are, President Dr. Geo. Watt; 1st Vice-President Dr. Geo. W. Keely; 2nd Vice-President B. F. Robinson; Recording Secretary H. A. Smith; Corresponding Secretary A. W. Maxwell, Treasurer M. DeCamp.

If the Association continues with the energy that characterizes it in the beginning it will certainly be productive of much good to the profession in the State. Long may it prosper.

